# Investigating the impact of COVID-19 related worries and loneliness on alcohol consumption: an ecological momentary assessment

**DOI:** 10.1007/s00406-024-01941-6

**Published:** 2024-11-27

**Authors:** Matthias Haucke, Andreas Heinz, Stephan Heinzel, Shuyan Liu

**Affiliations:** 1 Department of Psychiatry and Psychotherapy, Charité– Universitätsmedizin Berlin (Campus Charité Mitte), Charitéplatz 1, 10117 Berlin, Germany; 2 Clinical Psychology and Psychotherapy, Department of Education and Psychology, Freie Universität Berlin, Berlin, Germany; 3 German Center for Mental Health (DZPG), Berlin, Germany; 4 Departement of Educational Sciences and Psychology, Clinical and Biological Psychology, Technische Universität Dortmund, Dortmund, Germany

**Keywords:** Perceived social isolation, Drinking behavior, Ecological momentary assessment, Social restrictions, Multilevel autoregressive (AR) model, Autocorrelation, Day-to-day drinking, Temporal dynamics, Hierarchical mediation model

## Abstract

Adverse alcohol consumption is a major public health concern, which might have been further increased by the COVID-19 pandemic. In this study we investigated the impact of a lockdown stage on the association between alcohol consumption, loneliness, and COVID-19-related worries. We used smartphone-based Ecological Momentary Assessment (EMA) conducted during the COVID-19 pandemic in Germany. We recruited 280 participants from the general population, who experienced at least mild loneliness and distress due to the COVID-19 pandemic. We assessed daily alcohol intake, loneliness, and COVID-19-related worries every evening for 7 consecutive days across a no-lockdown [8th August 2020–1st November 2020] and lockdown stage [2nd November 2020–11th March 2021]. We did not find that a lockdown stage, compared to a no-lockdown stage, is associated with increased alcohol consumption. We found that loneliness, previous day drinking, and COVID-19-related worries were not associated with increased, but with decreased alcohol consumption. Moreover, COVID-19-related worries were more negatively associated with alcohol consumption during a no-lockdown stage compared to a lockdown stage. We found that the effect of COVID-19 related worries on alcohol consumption is mediated by loneliness. Our study suggests that heightened levels of worry can decrease alcohol intake. This association can be explained by loneliness: individuals who worry more are lonelier and thus less likely to engage in social drinking. However, during a lockdown stage, the negative association between worrying and drinking diminishes.

## Introduction

In Germany, approximately 75% of the general population consumed alcohol in the preceding 30 days and 3.1% are classified as alcohol-dependent [3]. Excessive alcohol consumption has severe health and socioeconomic consequences [26, 28]. The COVID-19 pandemic could have further increased alcohol consumption, yet, so far, the evidence on a pandemic impact on alcohol consumption is mixed [15, 17, 36, 44]. In this study we investigate whether a lockdown stage changes the association between alcohol consumption, state loneliness, and COVID-19-related worries.

There are mixed findings on COVID-19 pandemic on alcohol consumption, some studies reporting decreased [25], others reporting increased alcohol consumption [40, 42]. These mixed findings could be caused by averaged-based assessment of alcohol intake, which does not allow to examine the day-to-day fluctuation of alcohol consumption. Therefore, we used ecological momentary assessment (EMA), which allows to investigate the day-to-day fluctuation of state loneliness, COVID-19 worries and alcohol consumption [46].

In addition, previous studies were conducted during different stages of the COVID-19 pandemic and within different countries with various public health measures [55]. Thus, we investigated the underlying reasons of a possible COVID-19 impact on drinking habits. A potential COVID-19 stressor that is associated with alcohol consumption are COVID-19-related worries [11, 39]. Worries can be defined as “an attempt to engage in mental problem-solving on an issue whose outcome is uncertain but contains the possibility of one or more negative outcomes” [5]. Worries focuse on future potential threat and involve imagined catastrophes, uncertainties and risks [52]. Coping with one’s worries and to forget and escape one’s problems is an often reported reason for alcohol consumption [12, 38]. In line with this, studies prior to the pandemic indicate that worries can increase alcohol consumption [11, 39].

However, worrying about the impact of COVID-19 and associated lockdown measures may be rather maladaptive coping with the pandemic [37]. The COVID-19 pandemic and lockdown measures (e.g., business closure, lay-off, overstrained hospitals) may induce a passive focus on unchangeable causes, as the pandemic affects many uncontrollable macro-environment factors [12, 37]. Macro-environment factors are situational factors that feel outside of people’s control, including employment rates, one’s family health status or the country’s economic situation [8]. Compared to a no-lockdown, during a lockdown stage there are more distressing macro factors, thus we expect that COVID-19 related worries will be more positively associated with alcohol consumption.

Moreover, physical distancing measures to halt the spread of the virus are associated with an increase of loneliness [7, 9, 23, 33]. Loneliness can result from the perception that current relationships do not match desired relationships [2]. Loneliness can be a significant risk factor for alcohol abuse, yet the association between loneliness and alcohol consumption is inconsistent [1, 10]. A potential reason for this inconsistency is that studies often do not distinguish between social and solitary drinking [2]. In the case of social drinking, one would expect a negative association between loneliness and alcohol consumption. For example, people who spend most evenings out with their friends (i.e. people who are less lonely) report higher alcohol consumption [16, 50]. Moreover, the average daily alcohol intake is greater during leisure days than during workdays [27, 47]. On the other hand, in the case of solitary drinking one would expect a positive association between loneliness and alcohol consumption. Solitary drinking is associated with alcohol problems and drinking to cope with negative mood [34, 48]. Lockdown measures include the closing of public spaces (i.e., bars and clubs), physical distancing measures, and the fear of getting infected might made it harder or less motivating to engage in social drinking. Because of a possible increase in lonely drinking, we expect a more positive association between loneliness and alcohol consumption during a lockdown compared to a no-lockdown stage.

Moreover, a lockdown may change day-to-day fluctuation of alcohol consumption. We define daily fluctuations as the impact of alcohol consumption on one day on drinking behavior the following day. Identifying day-to-day alcohol consumption patterns can be used to distinguish consumption severity and to predict whether an alcohol intervention is effective [4, 49]. As alcohol consumption induces hangover symptoms, such as headache, tremulousness, nausea, diarrhea, and fatigue [53], alcohol consumption on one day is associated with less alcohol consumption on the next day (i.e. a negative autocorrelation). However, during times of lockdown, there are fewer chances to engage in pleasurable activities (e.g., meeting with friends, going to the cinema), which can help to cope with aversive feeling and worries. In addition, a lockdown increased psychological and physiological distress [19, 20, 29], which may introduce a higher motivation to drink to cope with negative emotional states. Thus, it may decrease one’s willingness to control one’s alcohol consumption despite hangover effects, leading to a stronger day-to-day effect of alcohol consumption during lockdown compared to no-lockdown.

**Fig. 1:**
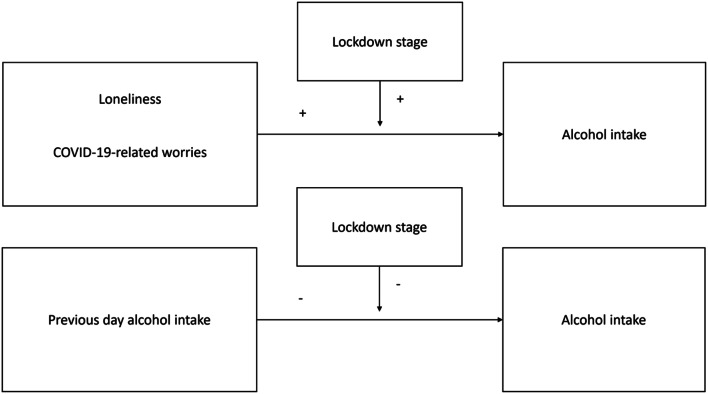
Summary of the hypotheses

In this study, we investigated how state loneliness and COVID-19 related worries affect alcohol consumption, during a no-lockdown and lockdown stage. We hypothesize the following: A lockdown stage, compared to a no-lockdown stage, will be positively associated with alcohol consumption. Moreover, COVID-19-related-worries and state loneliness are positively associated with alcohol consumption. Specifically, COVID-19-related worries and state loneliness are associated with higher alcohol consumption during lockdown compared to no-lockdown. Overall, drinking on one day is associated with drinking less the following day. However, drinking on one day is associated with more drinking the following day during a lockdown compared to no-lockdown stage (see Fig. 1).

## Methods

### Participants and procedure

**Fig. 2:**
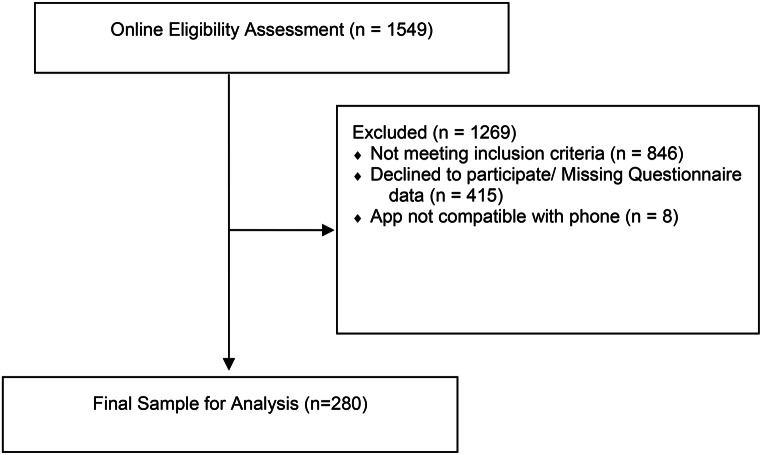
Recruitment flow

We screened 1549 participants for our ecological momentary study between August 2020 and March 2021. We recruited participants via advertisements on university websites, Twitter, and eBay classifieds. To screen participants, we used an online questionnaire on the Siuvo Intelligent Psychological Assessment Platform. 280 participants were eligible and gave informed consent to participant. The eligibility criteria were (1) aged 18 years or older, (2) not working a night shift, (3) using an Android Smartphone, (4) speaking fluent German, and sometimes felt lonely and mildly distressed according to the COVID-19 Peritraumatic Distress Index (CPDI; cutoff score = 28) and a short 8-item UCLA Loneliness Scale (ULS-8; cut-of score = 16) [22]. The detailed items of the CPDI an ULS-8 questionnaires can be found in our previous studies [30, 32]. The recruitment flow is shown in Fig. 2. The study was conducted in Germany during a no-lockdown stage (8 August – 1 November 2020) and a lockdown stage (2 November 2020–11 March 2021). An overview of the restrictions (e.g., social restriction, closing of restaurant, business shutdown) can be found in Supplement A. The study was approved by both Ethics Committee of Charité – Universitätsmedizin Berlin (ref: EA2/143/20) and Ethics Committee of Freie Universität Berlin (ref: 030/2020).

### Ecological momentary assessment

We used a smartphone application “movisensXS” (movisens GmbH, Karlsruhe, Germany) that is compliant with the General Data Protection Regulation (European Union) and Berlin Data Protection Act (Berliner Datenschutzgesetz – BlnDSG). The EMA consisted of a socio-demographic assessment (e.g., age, gender, and years of education) and repeated sampling of participants’ behaviors and experiences. Participants filled in questionnaires about their feelings of loneliness, alcohol consumption and COVID-19-related worries for 7 consecutive days in which they received 1 prompt (approximately at 10 p.m.). The EMA questionnaire lasted approximately 5 min. COVID-19-related worries was assessed on a visual analogue scale (0–100: 0 = not at all, 100 = extremely). State loneliness was measured with a short 3-item UCLA Loneliness Scale (ULS-3) [24], on a visual analogue scale (0–100: 0 = not at all, 100 = extremely). Daily alcohol consumption was assessed by asking, “How many alcoholic drinks did you have today?” Participants were shown examples of a standard drink of alcohol for each category (beer, wine, liquor, and cocktail). Options ranged from 0 (no drinks) to 10 standard drinks. One standard drink in Germany contains between 10 and 12 g of alcohol (on average 11 g) [35].

### Statistical analysis

We conducted our statistical analyses in the R version 4.1.0 system (www.r-project.org). To consider the hierarchical data structure and autoregressive parameters, we performed model selection using autoregressive (AR) multilevel models with the dependent variable alcohol consumption (*Model 1*). We followed the approach by Haan-Rietdijk et al. [14]; details about the model selection procedure can be found in the *Appendix* B. On average participants missed 0.35 days (SD = 0.83). Three participants were missing more than 4 days, and thus were excluded from the analysis.

We calculated a lag-1 two-level autocorrelation (AR) model, which allows to separate the variance of alcohol consumption into variance at the person level (level 2), and variance at the day level (level 1). That is, we created an autocorrelation variable of alcohol consumption (i.e., how much alcohol consumption on one day predicts alcohol consumption on the next day). The first response day of the week was excluded from the analysis to remove possible unexplained carryover effects resulting from the day before the measurement started. Model selection procedure resulted in a final model with a random effect “Day-to-day drinking”.

Furthermore, we added “Loneliness”, “COVID-19-related worries” as continuous predictors. We included “Lockdown” (“no-lockdown” and “lockdown”) as well as “Holiday” (i.e., Christmas on December 24, 2020, and New Years’ Eve on December 31, 2020) as categorical predictors. We used dummy coding to compare the mean of the outcome variable “Alcohol Consumption” for each level of the categorical variables (“Holiday”, “Lockdown”) with the mean at the reference group (“no holidays” and “ no-lockdown”) respectively. In addition, we added the interaction effects “Day-to-day Drinking” × “Lockdown”, “Loneliness” × “Lockdown”, and “COVID-19-related worries” × “Lockdown”. To counter problems with multicollinearity resulting from the added interaction term, and because we are interested in between-person effects, we centered each predictor involved in the interaction at their respective grand mean [18]. An overview of the used variables can be seen in Table 1.

**Table 1:** Variable overview and level in the hierarchical multiple regression. Level 1 measures are nested within level 2 measures. Grand mean is the overall mean of the specific variables across participants. The slope of a random effect varies across participants

Level 2 measures	Grand-mean centered	Fixed or random effect
Lockdown stage (i.e., lockdown, no-lockdown)	No	Fixed
**Level 1 measures**		
Holiday (i.e., Christmas, New Years’ Eve)	No	Fixed
Daily Loneliness	Yes	Fixed
Daily COVID-19 related worries	Yes	Fixed
Day-to-day drinking (i.e., autocorrelation of alcohol consumption)	Yes	Random

## Results

### Descriptives

280 participants in Germany completed in our EMA study. Sample characteristics are shown in Table 2.

**Table 2:** Demographics and sample characteristics

Variable	Group			Statistical analysis
	No-Lockdown (*N* = 137)	Lockdown (*N* = 143)	Total (*N* = 280)	t-test or chi-square test
Age (in years), mean (SD)	31.23 (10.61)	30.7 (12.03)	30.96 (11.33)	t = -0.39, (268.71), *p*-value = 0.70
Gender, n (%)	Female 85 (62%) Male 52 (38%) Diverse 0 (0%)	Female 105 (73%) Male 35 (24%) Diverse 3 (2%)	Female 190 (68%) Male 87 (31%) Diverse 3 (1%)	X-squared = 3.43(2), *p*-value = 0.18
Education (in years), mean (SD)	15.25 (3,76)	15.15 (3.68)	15.34 (3.84)	t = 0.43 (271.76) *p*-value = 0.67
ULS-8 Loneliness score, mean (SD)	21.9 (3.99)	23.27 (3.68)	22.59 (3.98)	t = 2.89 (271.36), *p*-value = 0.004
CPDI Distress index, mean (SD)	48.13 (16.06)	46.92 (13.19)	47.52 (14.67)	t = -0.68 (260.65), *p*-value = 0.50
Alcohol Consumption, mean (SD)	1.52 (0.78)	1.56 (0.76)	1.54 (0.77)	t = 0.37, (276.49), *p*-value = 0.7097
COVID-19-related Worries, mean (SD)	34.13 (21.2)	40.49 (20.13)	37.38 (20.87)	t = 2.574, (275.55), *p*-value = 0.01
Evening Loneliness, mean (SD)	22.63 (20.42)	22.58 (20.13)	22.6 (20.23)	t = -0.02 (277.1), *p*-value = 0.98

**Fig. 3:**
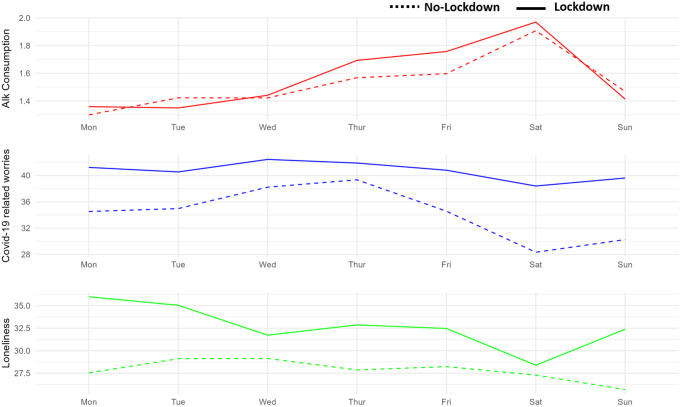
Average alcohol consumption, COVID-19 related worries as well as loneliness during each weekday, ranging from Monday to Sunday

As shown in Fig. 3, alcohol consumptions increased as the weekend approached. At the same time, on Fridays and Saturdays, there was a decrease in COVID-19-related worry and loneliness. Notably, the reduction in COVID-19-related worry was less pronounced during a lockdown period compared to a no-lockdown period. Furthermore, a stronger decrease in loneliness was observed as the weekend approached during a lockdown, compared to a no-lockdown.

### Main analysis

#### Results

**Fig. 4:**
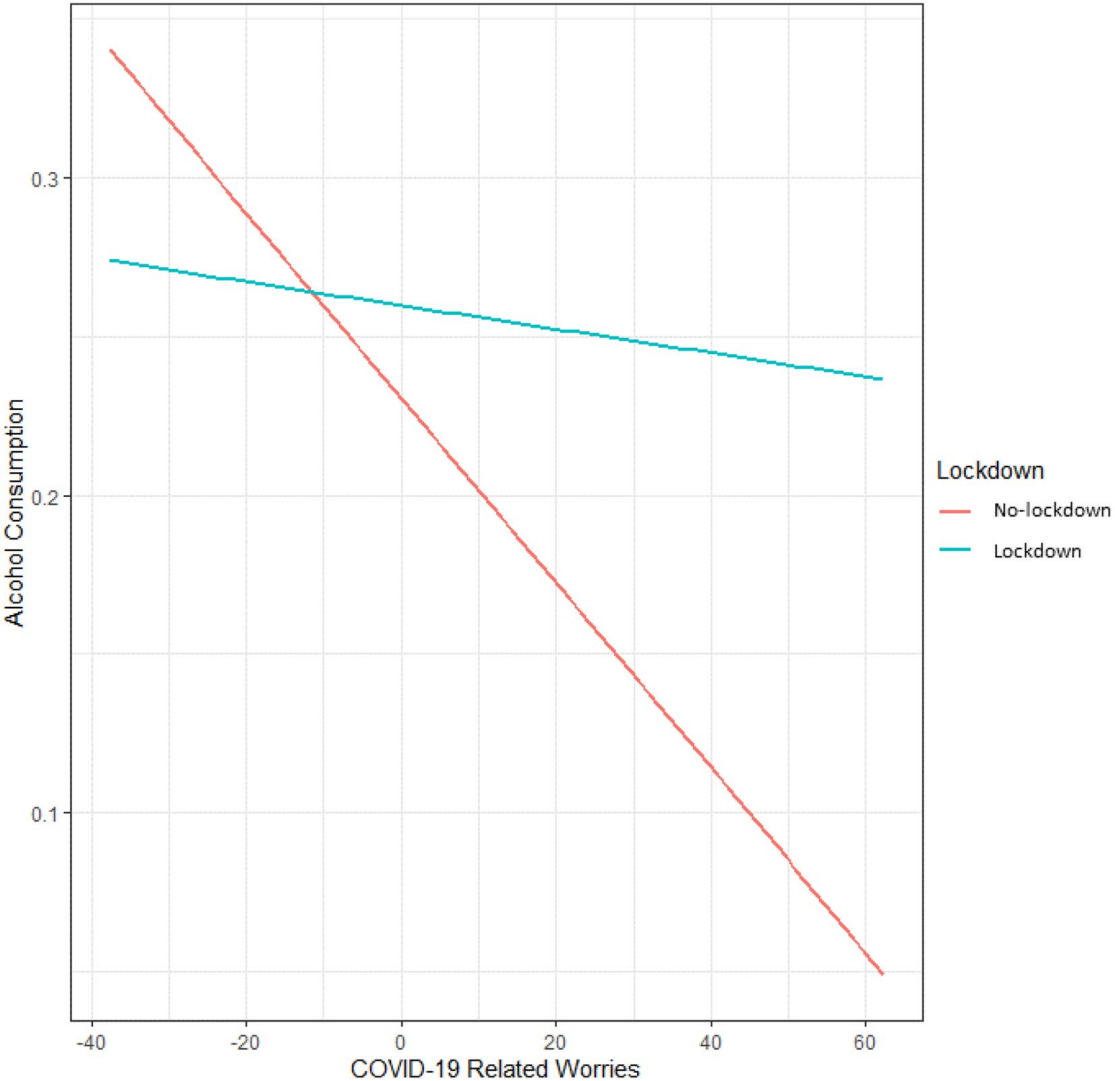
The association between COVID-19-related worries and alcohol consumption. Alcohol consumption is displayed as a function of COVID-19-related worries during no-lockdown (*N* = 137; red) versus lockdown (*N* = 143; blue). In both lockdowns stages there is a negative association between COVID-19 related worries and alcohol consumption. Interestingly, COVID-19 related worries are associated with higher alcohol consumption during a lockdown compared to a no-lockdown stage

We found that previous-day drinking (*b* = -0.11, *t*(160.70) = -2.40, *p* = 0.018), COVID-19 -related worries (*b* = -0.002, *t*(1189) = -2.93, *p* = 0.003) and state loneliness (*b* = -0.001, *t*(1214) = -2.06, *p* = 0.039) were significantly negatively associated with alcohol consumption. The interaction between COVID-19-related worries and lockdown stage (*b* = 0.003, *t*(1293) = 2.43, *p* = 0.016) were positively associated with alcohol consumption, as shown in Fig. 4. The other variable were not significantly associated (*p* > 0.05) with alcohol consumption, see Table 3.

**Table 3:** Multiple regression results with log alcohol consumption as the dependent variable. AIC = 2153.932, BIC = 2218.007, Pseudo-R² (fixed effects) = 0.033, Pseudo-R² (total) = 0.299

Variable	b	SE b	t value	*p* value
Previous day drinking	-0.112	0.047	-2.4	**0.018**
COVID-19-related worries	-0.002	0.001	-2.93	.**003**
Loneliness	-0.001	< 0.001	-2.06	**0.039**
Lockdown stage	0.032	0.039	0.84	0.404
Holiday	0.381	0.198	1.92	0.055
**Interaction effects**				
COVID-19 related worries × Lockdown stage	0.003	0.001	2.42	**0.016**
Loneliness × Lockdown stage	< -0.001	< 0.000	− 0.97	0.335
Previous day alcohol consumption × Lockdown stage	0.070	0.064	1.09	0.278

### Interim conclusion

Contrary to our hypothesis we found that state loneliness and COVID-19 related worries are not positively, but negatively associated with alcohol consumption. Moreover, we did not find increased alcohol intake during a lockdown compared to a lockdown stage. However, we found a negative autocorrelation effect of alcohol consumption (i.e., drinking on the previous day decreased drinking on the next day). In addition, we did not find an interaction effect between lockdown and loneliness, or lockdown stage and the autocorrelation of drinking. We found a significant interaction effect between lockdown and COVID-19 related worries. That is compared to a no-lockdown, during a lockdown, COVID-19 related worries are less negatively associated with alcohol consumption. In a mediation analysis we tested whether state loneliness could explain the negative association between COVID-19 related worries and alcohol consumption.

### Mediation analysis

We investigate whether state loneliness mediates the association between COVID-19-related worries and alcohol consumption. We used the multilevel mediation analysis (MLMed) module in SPSS Statistics Version 26 (SPSS Inc., Chicago, IL, USA) to perform a test for multilevel mediation [21]. We estimated the within and between group effects of each variable separately. We focused on the resulting within group indirect effect, as we are interested in the effect of COVID-19-related worries on alcohol consumption through state loneliness within individual participants. We calculated 95% Monte Carlo confidence intervals (MCCI) around the estimated within-group indirect effects [21]. Statistical significance was concluded when the MCCI did not include 0. The final mediation model includes a random intercept for the outcome (“Alcohol Consumption”), a random intercept for the mediator (“Loneliness”) and random slopes from “Loneliness” to “Alcohol Consumption”, as well as a random slope form “COVID-19 worries” to “Loneliness”. Details about the model selection procedure can be found in the Table 3 of *Appendix* B.

**Fig. 5:**
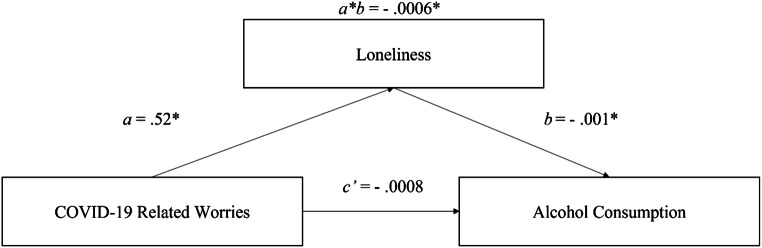
Within-group fixed effect to estimate the multilevel mediation model. All within-group unstandardized coefficients are statistically significant (*p* < 0.05). Path *c*’ describes the direct effect of COVID-19-related worries on alcohol consumption. Path *a* describes the effect of COVID-19-related worries on loneliness, path *b* describes the effect of loneliness on alcohol consumption. The within indirect effect of loneliness (-0.0006) is the product of **a** and **b**

To explore the negative impact of COVID-19-related worries on alcohol consumption, we tested whether state loneliness mediates the relation between COVID-19-related worries and alcohol consumption. With 10,000 Monte Carlo replications, the results did show a significant negative indirect within-group relationship between state loneliness and alcohol consumption via COVID-19-related worries (indirect effect = − 0.0006, MCCI = [-0.0010, − 0.0003]). That is on average, within a given participant, one unit increase of COVID-19 related worries results in 0.0006 units decrease in alcohol consumption through loneliness. The unstandardized coefficients “*b*” for the within-group effects are presented in Fig. 5.

## Discussion

Adverse alcohol consumption is a major public health and socio-economic concern worldwide, which might have been further exacerbated by the COVID-19 pandemic and related lockdown measures. In this study, we investigated how COVID-19 lockdown impacts the association between worry, loneliness and alcohol intake. Contrary to our hypothesis, we did not find an increase of alcohol consumption during a lockdown stage. Moreover, we found that state loneliness is negatively associated with alcohol consumption, independent of a lockdown stage. This suggest that drinking still occurs in social contexts even in times of physical distancing. Surprisingly, we found that worrying is negative associated with alcohol intake. Our study indicates that people who worry more, are more likely to be lonely and therefore less likely to engage in social drinking. Moreover, we found that COVID-19-related worries were less negatively associated with alcohol consumption during a lockdown stage.

During a lockdown stage there might be a less negative association between COVID-19-related worries and alcohol consumption, because of situational factors inducing different types of repetitive negative thinking and reasons to drink. Previous studies indicate two central forms of repetitive negative thinking: pondering and brooding [51]. While pondering reflects “a purposeful turning inward to engage in cognitive problem solving”, brooding reflects “a passive comparison of one’s current situation with some unachieved standard [53] (p. 256). Brooding might be more maladaptive form of coping with negative affect than reflection, as brooding leads to increased alcohol consumption [54]. Furthermore, the effect of worries on alcohol consumption depend on the situational context [52]. We measured during a lockdown stage in Germany, which imposed physical distancing measures, business shutdown, a loss of jobs along the increasing fear of becoming infected. These factors are outside people’s personal control. Therefore, worries in times of COVID-19 may resemble rather passive and maladaptive forms of repetitive negative thinking, such as brooding, which can lead to an increase in alcohol consumption [54]. A loss of control over pandemic stressors may lead to less social drinking and more “drinking-to-cope” to reduce the negative effects of worries [13]. Conversely, the diminishing sense of control over one’s concerns could have reduced the efficacy of social drinking as a means to alleviate worries.

Our study indicates that the negative association between COVID-19 related-worries and alcohol intake was mediated by loneliness. Worrying occurred in a solitary context, yet people drank in a social context. Thus, state loneliness caused by a lack of social contacts [7], may explain why worrying is associated with less drinking. We found that state loneliness was associated with decreased alcohol consumption. In line with this finding, studies before the COVID-19 pandemic showed that loneliness decreases drinking, which is strongly tied to a social context [2, 6, 41]. Thus, our results indicate that people engaged in social drinking rather than drinking to cope with worries, yet this effect was less pronounced during a lockdown stage.

Moreover, we found that people did not consume more alcohol during a lockdown than a no-lockdown stage. This contrast earlier studies, which found a decline in alcohol consumption during the first months of COVID-19-related social restrictions [25], hypothetically due to reduced availability and affordability of alcohol and fewer chances to socialize onsite [43]. These contrasting finding might be explained by different pandemic stages. We measured alcohol consumption during the second wave of the pandemic in winter 2020/2021, during which people might have already adapted their drinking habits to lockdown measures.

Day-to-day alcohol consumption patterns can be used to further distinguish between heavy or light drinking patterns [4, 49]. We found that alcohol consumption on one day is negatively associated with alcohol consumption on the next day. Contrary to our hypothesis, we did not find that this temporal effect of alcohol consumption changed under a lockdown. Therefore, we did not find evidence that people are less able or unwilling to control their alcohol consumption during lockdown.

### Limitation and future studies

Our current results are limited by the lack of comparison between drinking alone and drinking with others, virtual drinking and in-person drinking. Furthermore, we conducted a study involving a vulnerable population that is particularly at risk of developing mental health issues during the COVID-19 pandemic. During lockdown stringent measures were imposed, such as physical distancing, business closures, widespread job losses, and an increased fear of infection, creating an unprecedented situation. Additionally, we specifically targeted individuals who reported experiencing at least mild distress and loneliness, which accounted for approximately 45% of our recruited participants. Together these factors may limit the generalizability of our findings. In addition, we did not assess the prevalence of alcohol use disorder or other relevant psychiatric diagnosis in our sample. We did not measure alcohol consumption after 10 pm. Moreover, we gathered data between two lockdown stages, where the first lockdown stage phase [March to May 2020] occurred before the measured no-lockdown period [August to November 2020]. As a result, the effects observed during the no-lockdown stage could have been influenced by the preceding lockdown, such fear of another lockdown, fear of getting infected or job loss. Another limitation is the reliance on self-reported data, which may be affected by memory biases or a tendency for socially desirable responses. Finally, since we conducted an EMA study, our sample size was relatively small compared to traditional surveys. However, each participant contributed a rich set of observations, offering valuable insights at the individual level and enabling us to estimate temporal effects.

Our results hint at ways to investigate effective alcohol interventions that target a combination of multiple factors, includin repetitive negative thinking. These combined factors may be warning signals of potentially increased alcohol intake and could inform the triggering of ecological momentary interventions (EMI) [31, 45]. Future studies may further investigate the context in which drinking occurs, that is, social and solitary drinking likely have distinct mechanisms and consequences.

## Conclusion

Adverse alcohol consumption is a major public health concern, potentially exacerbated by the COVID-19 pandemic. However, we did not find that a lockdown stage was associated with alcohol consumption. We found that COVID-19-related worries, drinking on the previous day and state loneliness were negatively associated with drinking. Our study highlights that individuals who worry more, are more likely to experience loneliness, and therefore are less likely to engage in social drinking. In addition, COVID-19 related worries were less negatively associated with alcohol consumption during a lockdown stage. This suggests that, during a lockdown, people might have engaged less in social drinking, or social drinking did less effectively alleviate worries.

## Data Availability

Data and analysis code will be made available online on the open science framework after publication: https://osf.io/w6pxb/
