# Correction: Investigating the impact of COVID-19 related worries and loneliness on alcohol consumption: an ecological momentary assessment

**DOI:** 10.1007/s00406-025-01957-6

**Published:** 2025-01-17

**Authors:** Matthias Haucke, Andreas Heinz, Stephan Heinzel, Shuyan Liu

**Affiliations:** 1 Department of Psychiatry and Psychotherapy, Charité–Universitätsmedizin Berlin (Campus Charité Mitte), Charitéplatz 1, 10117 Berlin, Germany; 2 Clinical Psychology and Psychotherapy, Department of Education and Psychology, Freie Universität Berlin, Berlin, Germany; 3 German Center for Mental Health (DZPG), Berlin, Germany; 4 Departement of Educational Sciences and Psychology, Clinical and Biological Psychology, Technische Universität Dortmund, Dortmund, Germany

**Correction: European Archives of Psychiatry and Clinical Neuroscience**

10.1007/s00406-024-01941-6

In the sentence beginning ‘We found that previous-day drinking…’ under section Results inMain Analysis in this article, the value ‘COVID-19 -related worries (*b* = − 0.02, *t* (1189) = − 2.93, *p* = 0.003)’ should have read ‘COVID-19 -related worries (*b* = − 0.002, *t* (1189) = − 2.93, *p* = 0.003)’.

The corrected sentence should read as follows:

We found that previous-day drinking (*b* = − 0.11, *t* (160.70) = − 2.40, *p* = 0.018), COVID-19 -related worries (*b* = − 0.002, *t* (1189) = − 2.93, *p* = 0.003) and state loneliness (*b* = − 0.001, *t* (1214) = − 2.06, *p* = 0.039) were significantly negatively associated with alcohol consumption.

The original article has been corrected.

